# ASB2 is a novel E3 ligase of SMAD9 required for cardiogenesis

**DOI:** 10.1038/s41598-021-02390-0

**Published:** 2021-11-29

**Authors:** Kyung-Duk Min, Masanori Asakura, Manabu Shirai, Satoru Yamazaki, Shin Ito, Hai Ying Fu, Hiroshi Asanuma, Yoshihiro Asano, Tetsuo Minamino, Seiji Takashima, Masafumi Kitakaze

**Affiliations:** 1 Department of Clinical Research and Development, National Cerebral and Cardiovascular Center, 6-1 Kishibe- Shimmachi, Suita, Osaka 564-8565 Japan; 2 Department of Cardiovascular and Renal Medicine, Hyogo College of Medicine, Hyogo, Japan; 3 Department of Bioscience, National Cerebral and Cardiovascular Center, Osaka, Japan; 4 Omics Research Center, National Cerebral and Cardiovascular Center, Osaka, Japan; 5 Department of Cell Biology, National Cerebral and Cardiovascular Center, Osaka, Japan; 6 Department of Molecular Pharmacology, National Cerebral and Cardiovascular Center, Osaka, Japan; 7 Department of Cardiorenal and Cerebrovascular Medicine, Faculty of Medicine, Kagawa University, Kagawa, Japan; 8 Department of Internal Medicine, Meiji University of Integrative Medicine, Kyoto, Japan; 9 Department of Cardiovascular Medicine, Osaka University Graduate School of Medicine, Osaka, Japan; 10 Department of Medical Biochemistry, Osaka University Graduate School of Medicine, Osaka, Japan; 11 Hanwa Daini Senboku Hospital, Sakai, Osaka Japan

**Keywords:** Differentiation, Ubiquitylated proteins

## Abstract

Cardiogenesis requires the orchestrated spatiotemporal tuning of BMP signalling upon the balance between induction and counter-acting suppression of the differentiation of the cardiac tissue. SMADs are key intracellular transducers and the selective degradation of SMADs by the ubiquitin–proteasome system is pivotal in the spatiotemporal tuning of BMP signalling. However, among three SMADs for BMP signalling, SMAD1/5/9, only the specific E3 ligase of SMAD9 remains poorly investigated. Here, we report for the first time that SMAD9, but not the other SMADs, is ubiquitylated by the E3 ligase ASB2 and targeted for proteasomal degradation. ASB2, as well as Smad9, is conserved among vertebrates. ASB2 expression was specific to the cardiac region from the very early stage of cardiac differentiation in embryogenesis of mouse. Knockdown of *Asb2* in zebrafish resulted in a thinned ventricular wall and dilated ventricle, which were rescued by simultaneous knockdown of *Smad9*. Abundant Smad9 protein leads to dysregulated cardiac differentiation through a mechanism involving Tbx2, and the BMP signal conducted by Smad9 was downregulated under quantitative suppression of Smad9 by Asb2. Our findings demonstrate that ASB2 is the E3 ligase of SMAD9 and plays a pivotal role in cardiogenesis through regulating BMP signalling.

## Introduction

Bone morphogenetic protein (BMP) signalling is one of the most fundamental signals for embryogenesis, as it regulates a wide variety of the biological reactions during development of various organs. The BMP signalling is activated when a ligand binds to dimerized type II and type I receptor serine/threonine protein kinases. Consequently, SMAD1/5/9 (SMAD9 is also known as SMAD8), the receptor-regulated SMAD transcription factors (R-SMADs) are phosphorylated at their C-terminal SXS motif to form a heteromeric complex with common SMAD4 (Co-SMAD). Then, these complexes translocate into the nucleus to regulate the expression of hundreds of target genes of BMP signalling as transcription factors.

In the embryonic development, BMP signalling is required for apoptotic cell death which enables appropriate remodelling and morphogenesis of various organs^1–3^. Intriguingly, a growing body of evidence indicates that not only activation but also inhibition of BMP signalling is required for the induction of various tissues in the developing embryo. For example, the inhibition of extracellular BMP ligand activity by either Noggin or Chordin, historically known as Spemann’s organizers, is crucial for neural induction^4–7^. Conversely, prolonged exposure to BMP signalling after cardiac mesoderm formation attenuates the differentiation of cardiac progenitor cells into cardiomyocytes^8,9^. Excessive BMP signalling or loss of antagonizing mechanisms result in cell death and apoptosis in various organs including heart^10–12^, indicating that antagonizing BMP signalling at the proper phase is essential for normal tissue development.

Among the various antagonizing mechanisms that have evolved to enable dynamic tuning of the duration and magnitude of the BMP signalling, the activities of R-SMADs, as the critical intracellular transducers of BMP signalling, are strictly regulated in various manners. On the cell membrane, ligand-dependent R-SMAD phosphorylation is inhibited by BMP and activin membrane-bound inhibitor homologue (BAMBI) through sequestering the ligands from the receptors^13^. DRAGON, another membrane protein, is also known to attenuate BMP signalling by inhibiting ligand-receptor binding required for R-SMAD activation^14–16^. Inside cells, SMAD6 and SMAD7, the inhibitory SMADs, interact with R-SMADs to interfere with the formation of the heteromer with SMAD4 required for the translocation to the nucleus^17^. In addition to these functional regulations, R-SMADs are quantitatively regulated by active, selective, and temporal degradation by the ubiquitin–proteasome system (UPS). Previous studies reported that the specific recruitment of R-SMADs to the UPS is enabled by the HECT type E3 ligase Smurf 1/2^18–20^. However, Smurf1/2 has been shown to ubiquitylate SMAD1 and SMAD5 but not SMAD9^21,22^. Among the three R-SMADs involved in BMP signalling, SMAD9 is the only one for which no specific E3 ligase has been found.

The previous reports indicated the unique properties of SMAD9 that is independent from SMAD1/5. Overexpression of Smurf1 in the murine embryonic lung negatively regulates lung branching through specific reduction of Smad1 and Smad5 but not Smad9^23^. Another study demonstrated that forced expression of Smad9 mediated by mRNA injection into zebrafish embryos leads to abnormal ventralization^24^. These lines of evidence indicate that SMAD9 has a unique biological function and that SMAD9 may have its specific E3 ligase in the tissue where BMP signalling needs to be regulated.

Here, we introduce a novel E3 ligase for SMAD9, Ankyrin repeat and SOCS box containing 2 (ASB2), which is required for normal cardiac development. ASB2 is specifically expressed in the cardiac region as early as from cardiac crescent formation in the early developmental stage. ASB2 ubiquitylates SMAD9 but not SMAD1 or SMAD5 and targets SMAD9 for proteasomal degradation to avoid excessive accumulation of SMAD9 in the embryo so that normal cardiac development is achieved. Our results suggest that ASB2 plays a crucial role in BMP signal regulation during cardiac development through quantitative regulation of SMAD9.

## Results

### Identification of ASB2 as an interactor and E3 ligase of SMAD9

**Figure 1:**
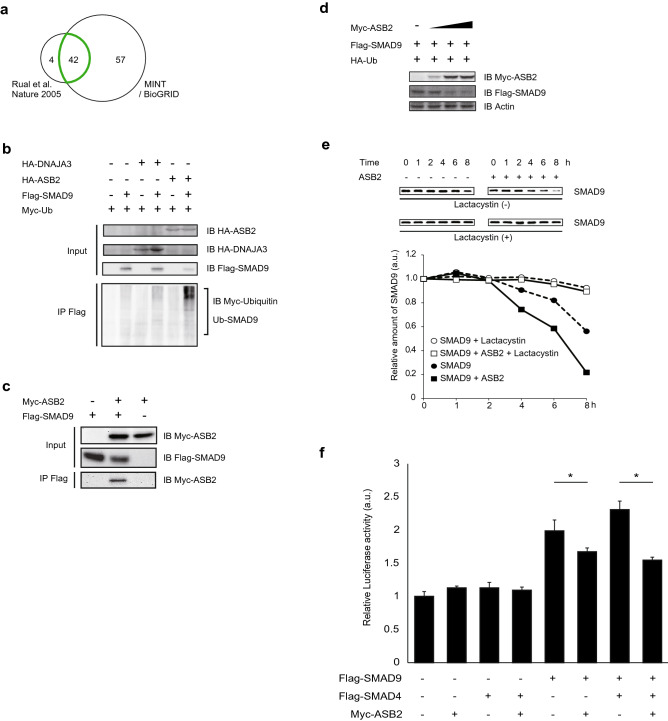
ASB2 is a novel E3 ligase of SMAD9. (**a**) Independent databeses of interactome were used to narrow the candidates for SMAD9-binding proteins. Finally, 42 proteins were remained (see Table1). (**b**–**d**) HEK293T cells were transfected with vectors encoding indicated tagged proteins and Flag-immunoprecipitates or lysates were separated using SDS-PAGE and immunoblotted with the indicated antibodies. (**b**) ASB2 but not DNAJA3 ubiquitilated SMAD. (**c**) ASB2 interacts SMAD9. (**d**) The amounts of transfected vector encoding Myc-Asb2 were 0, 0.1, 1, and 2 µg, respectively (from the left column) The total amount of the transfected vector was adjusted using empty vector. The protein amount of SMAD9 was negatively correlated with that of ASB2. (**e**) Pulse chase analysis. To terminate further protein synthesis, cells were treated with 50 µg/mL cycloheximide with or without 10 µM lactacystin at 24 h after transfection. Total cell lysates were obtained at the indicated times and subjected to western blot analysis. Protein levels were determined using densitometry. (**f**) Asb2 suppress BMP signalling through degradation of Smad9. HeLa cells were co-transfected with Xvent2-Luc plasmid, which expresses luciferase under BMP signalling, and a pGL4.75 vector as a control, which constantly expresses *Renilla* luciferase, and vectors encoding Flag-SMAD9, Flag-SMAD4 and Myc-ASB2 as indicated. Cells were treated with 100 µM BMP2. Using cell lysates obtained 24 h after transfection, the luminescence of both firefly and *Renilla* luciferase were measured. Luciferase activity under BMP signalling was normalized with *Renilla* luciferase activity. The cropped gel images were delineated and uncropped gel images are shown in the Supplementary Information. The data are shown as the mean ± s.d. Statistical analysis was performed using one-way ANOVA with Tukey’s multiple comparison tests. *p < 0.05; ns, not significant.

To determine the novel regulatory mechanism of BMP signalling through quantitative regulation of SMAD9, we firstly searched for the potent E3 ligase of SMAD9. Because it was difficult and unreliable to search for the E3 ligase of a specific protein in a comprehensive fashion, we surveyed the in silico databases of the interactome to identify SMAD9-binding proteins (Fig. 1a). We conducted the in silico search using the major web-based interactome databases Molecular INTeraction (MINT; https://mint.bio.uniroma2.it/) and BioGRID (http://thebiogrid.org) and selected 99 protein candidates that possibly interact with SMAD9 (Supplementary Table S1–S3). Additionally, to narrow down the candidates, we referred to a previous study of the human interactome by Rual et al^25^ that employed yeast two hybrid assays. Ultimately, we found 42 possible binding proteins for SMAD9 (Table 1). For each of these candidates, we then conducted a thorough literature search and found that 2 proteins, namely DnaJ heat shock protein family (Hsp40) member A3 (DNAJA3) and ASB2, were reported to have E3 activity. To confirm whether these proteins bind and ubiquitylate SMAD9, we performed co-immunoprecipitation experiments and found that ubiquitylated SMAD9 was detected only in the presence of ASB2 (Fig. 1b), but not of DNAJA3. Smad9 was not ubiquitinated by the mutant Asb2 that has a single amino acid mutation on the functional motif of Asb2 that inhibit the formation of E3 ligase complex (Supplementary Fig. S1). These results demonstrate that ASB2, but not DNAJA3, is required for the ubiquitilation of SMAD9.

**Table 1:** Candidates of SMAD9 interacting proteins and their E3 activity.

Entrez GeneID	Gene symbol	Gene name	E3 activity
60	ACTB	Actin beta	No
160	AP2A1	Adaptor related protein complex 2 alpha 1 subunit	No
1442	CSH1	Chorionic somatomammotropin hormone 1	No
1583	CYP11A1	Cytochrome P450 family 11 subfamily A member 1	No
1877	E4F1	E4F transcription factor 1	No
2313	FLI1	Fli-1 proto-oncogene, ETS transcription factor	No
2512	FTL	Ferritin	No
2896	GRN	Granulin	No
3646	EIF3E	Eukaryotic translation initiation factor 3 subunit E	No
4245	MGAT1	Mannosyl (alpha-1,3-)-glycoprotein beta-1,2-N-acetylglucosaminyltransferase	No
5467	PPARD	Peroxisome proliferator-activated receptor delta	No
5660	PSAP	Prosaposin	No
5714	PSMD8	Proteasome 26S subunit, non-ATPase 8	No
5859	QARS	Glutaminyl-tRNA synthetase	No
6904	TBCD	Tubulin folding cofactor D	No
7266	DNAJC7	DnaJ (Hsp40) Homolog, Subfamily C, Member 7	No
8544	PIR	Pirin	No
8663	EIF3C	Eukaryotic translation initiation factor 3 subunit C	No
8665	EIF3F	Eukaryotic translation initiation factor 3 subunit F	No
9070	ASH2L	ash2 (absent, small, or homeotic)-like (Drosophila)	No
9093	DNAJA3	DnaJ (Hsp40) Homolog, Subfamily A, Member 3	Yes
11034	DSTN	Destrin	No
22943	DKK1	Dickkopf WNT signaling pathway inhibitor 1	No
23462	HEY1	Hes related family bHLH transcription factor with YRPW motif 1	No
23650	TRIM29	Tripartite motif containing 29	No
26508	HEYL	hes related family bHLH transcription factor with YRPW motif-like	No
29979	UBQLN1	Ubiquilin 1	No
51523	CXXC5	CXXC Finger Protein 5	No
51676	ASB2	Ankyrin repeat and SOCS box containing 2	Yes
55236	UBA6	Ubiquitin like modifier activating enzyme 6	No
55577	NAGK	N-Acetylglucosamine Kinase	No
55611	OTUB1	OTU Deubiquitinase, Ubiquitin Aldehyde Binding 1	No
56893	UBQLN4	Ubiquilin 4	No
57104	PNPLA2	Patatin like phospholipase domain containing 2	No
64129	TINAGL1	Tubulointerstitial nephritis antigen like 1	No
64333	ARHGAP9	Rho GTPase activating protein 9	No
64374	SIL1	SIL1 nucleotide exchange factor	No
64795	RMND5A	Required for meiotic nuclear division 5 homolog A	No
79048	SECISBP2	SECIS Binding Protein 2	No
79230	ZNF557	Zinc finger protein 557	No
80325	ABTB1	Ankyrin repeat and BTB (POZ) domain containing 1	No
81570	CLPB	ClpB homolog, mitochondrial AAA ATPase chaperonin	No

The potent interacting proteins of SMAD9 were narrowed down using 3 individual databases of interactome. MINT and BioGRID are common database of protein interaction provided on the web. Rual et al. provided human interactome database in the previously published study (see reference^19^). The thorough literature search revealed that, among these candidates, only DNAJA3 and ASB2 have the E3 activity.

### ASB2 specifically ubiquitylates SMAD9 and targets it for proteasomal degradation

Because of the conserved amino acid sequence of SMAD1/5/9 (Supplementary Fig. S2), we could not achieve an antibody that could effectively and specifically react only with SMAD9. Thus, we used tagged constructs to verify the physical interaction and E3 activity of ASB2 for SMAD9 using a co-IP assay. The HEK293T cells were transfected with vectors expressing Myc-tagged ASB2 (Myc-ASB2) and Flag-tagged SMAD9 (Flag-SMAD9). Twenty-four hours after the transfection, co-IP detected binding between ASB2 and SMAD9 (Fig. 1c). Next, we investigated whether ASB2 regulates protein levels of SMAD9 through UPS dependent degradation. Co-expression of ASB2 revealed a significant and dose-dependent decrease in SMAD9 (Fig. 1d). It should be noted that, in this assay, lesser amount of SMAD9 plasmid was used than in the ubiquitilation assay so that decreases in protein quantity can be detected more sensitively. Next, we investigated whether the decrease of SMAD9 protein induced by ASB2 is mediated by the proteasomal pathway. To test this hypothesis, cells were transfected with Flag-SMAD9 with or without Myc-ASB2 and treated with cycloheximide at 24 h after transfection to terminate further protein synthesis. Whole cell lysates collected in the subsequent time course were subjected to SDS-PAGE to measure the amount of protein. Our results showed that SMAD9 levels were slightly decreased within 8 h when expressed alone, but the amount of the decrease was accelerated with co-expression of ASB2. Importantly, this accelerated decrease was substantially reduced by co-incubation with a proteasome inhibitor, lactacystin (Fig. 1e). These results suggest that Asb2 interact with Smad9 and ubiquitilate it as E3 ligase in order to target Smad9 for proteasomal degradation. To confirm that ASB2-mediated degradation of SMAD9 affects BMP signalling, we quantified in vitro BMP signalling employing Xvent2-Luc plasmid, in which Xvent2 promoter containing the BMP response element is flanked by the luciferase gene. (Fig. 1f). Under stimulation with BMP2, Luciferase activity was strengthened by SMAD9 expression, but significantly suppressed under coexistence of ASB2. Co-expression of SMAD4 further strengthened the luciferase activity by SMAD9, and again, it was suppressed by coexistence of ASB2. Either ASB2 or SMAD4 expression alone did not affect the luciferase activity. The luciferase activity by Smad9 was not suppressed by loss-of-function mutant Asb2 (Supplementary Fig. S5). These results showed that BMP signalling conducted by SMAD9 is controlled by ASB2 through down-regulation of SMAD9.

### SMAD9 is specifically ubiquitylated by ASB2

**Figure 2:**
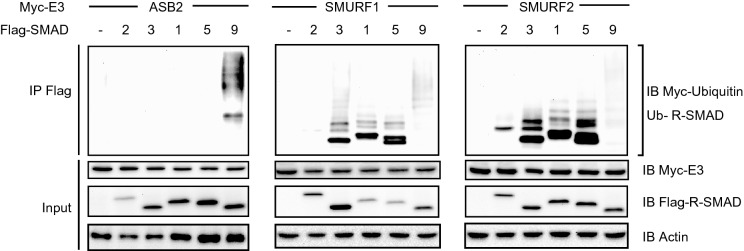
ASB2 specifically ubiquitylates SMAD9. HEK 293T cells were co-transfected with vectors coding HA-Ub, Myc-tagged E3 (Myc-ASB2, SMURF1 and SMURF2) and Flag-tagged R-SMADs (Flag-SMAD2, 3, 1, 5, 9) as indicated. Flag-immunoprecipitates or lysates were separated with SDS-PAGE and immunoblotted with the indicated antibodies. The cropped gel images were delineated and uncropped gel images are shown in the Supplementary Information.

Conventionally, SMURF 1/2 have been well investigated as the E3 ligases that target R-SMADs of the TGFβ superfamily for proteasomal degradation, resulting in negative regulation of signal transduction^18–20^. However, direct evidence of the SMAD9 ubiquitylation by SMURF 1/2 has not been reported. Thus, we compared the E3 activity of ASB2 and SMURF1/2 for every R-SMAD including Smad2/3 that work for TGFβ signalling pathway. Flag immunoprecipitation of whole cell lysate showed that ASB2 specifically ubiquitylated SMAD9 but no other R-SMADs. Consistent with previous reports, SMURF1 and SMURF2 ubiquitylated SMAD3 and SMAD1/5, and SMAD2/3 and SMAD1/5, respectively, but they showed quite weak E3 activity for SMAD9 (Fig. 2). These results indicate that the ASB2 is the unique E3 ligase of SMAD9, whilst the other R-SMADs share SMURF1/2 as their common E3 ligase.

### ASB2 is expressed specifically in heart during early developmental stages

In the embryogenesis, where BMP signalling plays crucial roles, the spatial regulation of Smad9 expression has been previously reported. Arnold et al. demonstrated that murine embryonic expression of *Smad9* begins after embryonic day 7.5 (E7.5) around the cardiac region and is lower in the tail bud region and allantois^26^. We hypothesized that *Asb2* is spatiotemporally co-expressed with Smad9 in the developing murine embryo.

**Figure 3:**
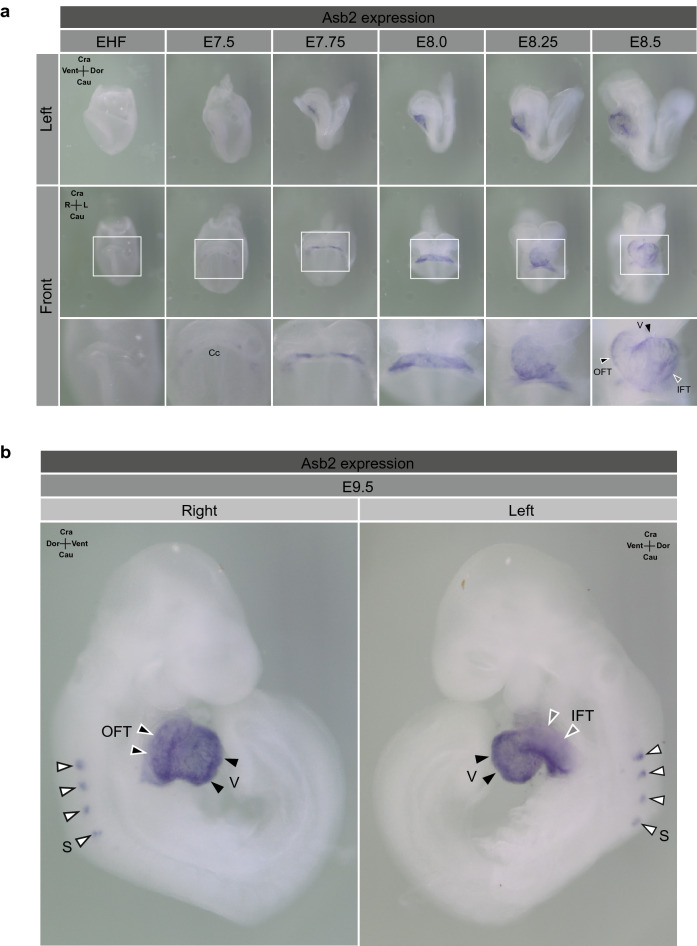
*Asb2* is specifically expressed in the cardiac region during early embryogenesis of mice. Whole mount in situ hybridization was performed using murine embryos. (**a**) *Asb*2 expression was detected in the cardiac region as early as formation of the cardiac crescent at E7.5. Thereafter, *Asb*2 expression was localized at the cardiac region until cardiac formation was completed. (**b**) At E9.5, *Asb2* expression was detected in the ventricular myocardium and outflow and inflow tracts. Additionally, extra-cardiac expression of *Asb2* was initiated at several somites at this stage. EHF, early head fold; Cc, cardiac crescent; OFT, outflow tract; IFT, inflow tract; v, ventricle; s, somite.

To test this hypothesis in detail, we first performed whole-mount in situ hybridization in murine embryos and investigated the spatiotemporal expression of *Asb2* in the developing embryo. Intriguingly, our results showed that the *Asb2* transcript was specifically detected in the cardiac region as early as the appearance of the cardiac crescent at E7.5 (Fig. 3a). Thereafter, *Asb2* expression was specifically detected in the cardiac region until heart formation was completed and was followed by slight expression in somites from E9.5 (Fig. 3b). Consistent with this data, RT-PCR using Multiple Tissue Panel (Takara Bio, Japan) revealed that ASB2 expression was detected mainly in heart and skeletal muscle among various tissues of adult and fetal human as well as mouse (Supplementary Fig. S2). Additionally, Asb2 and Smad9 were up-regulated in cardiomyocyte induction of both P19CL6 cells, which is known to differentiate into cardiomyocyte when cultured in presence of dimethylsulfoxide, and ES cells (Supplementary Fig. S3). These data suggest that *Asb2* is predominantly expressed in the developing heart from a very early stage of cardiogenesis. Highly specific expression of both Asb2 and Smad9 in the heart during embryonic development, along with the biochemical analysis showing the degradation of Smad9 specifically enabled by Asb2 (Fig. 2), suggests that Asb2 participates in cardiac development through interaction with, and quantitative regulation of, Smad9.

### ASB2 is essential for normal cardiac development in zebrafish embryos

**Figure 4:**
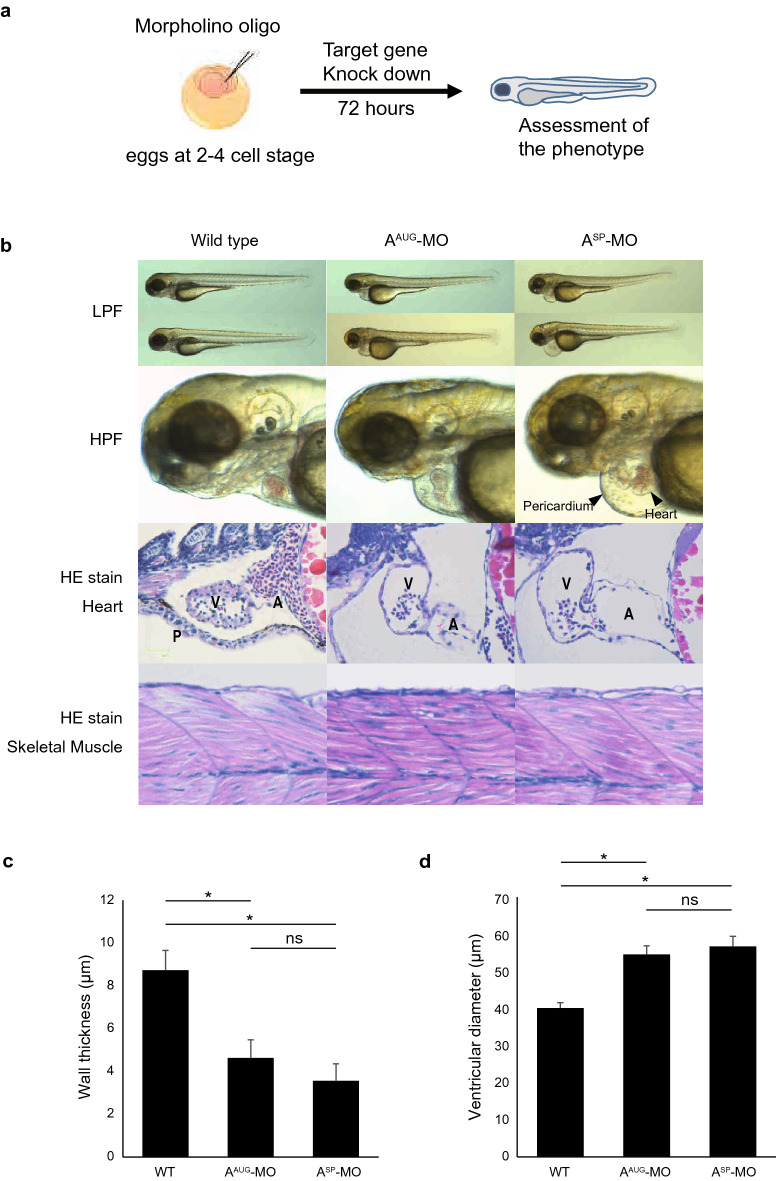
Knockdown of *Asb2* attenuates normal cardiac development. The representative phenotype of zebrafish injected with MO targeting *Asb2*. (**a**) Scheme of the zebrafish experiment. Morpholino oligo designed for targeted gene knock down was injected to the eggs of zebrafish at 2–4 cell stage. At 72 hpf (hours post fertilization), phenotype was assessed. (**b**–**d**) MO targeting either the start codon (A^AUG^-MO) or the splice donor and acceptor site of exon 7 (A^SP^-MO) of *Asb2* was injected into zebrafish embryos. At 72 hpf, both A^AUG^-MO and A^SP^-MO injection produced a phenotype of cardiac swelling (**b**). In histological analysis, thinner ventricle walls (**c**) and dilated ventricles (**d**) were observed in the A^AUG^- and A^SP^-MO injected groups compared to wild type siblings. In each group 5 to 7 animals were analysed. Note that no obvious change in phenotype was observed except in the heart. The data are shown as the mean ± SD. Statistical analysis was performed using one-way ANOVA with Tukey’s multiple comparison tests. *p < 0.05; ns, not significant; LPF, low power field; HPF, high power field; HE, Hematoxylin eosin; V, ventricle; A, atrium; P, pericardium.

**Figure 5:**
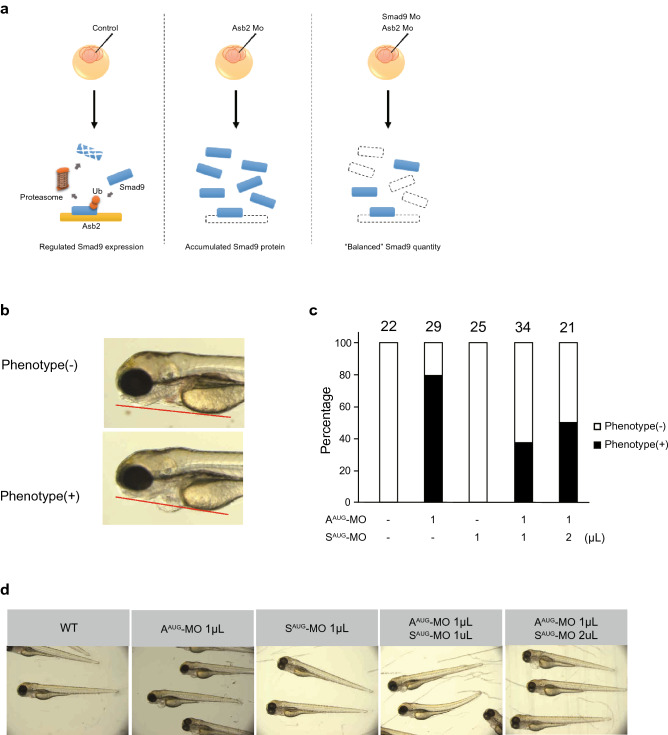
The phenotype of *Asb2* knockdown is rescued by simultaneous knockdown of *Smad9.* (**a**) Scheme of the experimental hypothesis. The balanced amount of Smad9 protein is enabled by expression as well as degradation of Smad9. The injection of Asb2 Mo may result in loss of active degradation of Smad9 protein leading to the accumulation of Smad9. Simultaneous knockdown of Asb2 and Smad9 may attenuate the accumulation of Smad9 protein. Therefore, MO targeting the start codon of *Asb2* (A^AUG^-MO) and/or *Smad9* (S^AUG^-MO) was injected into zebrafish embryos at the 2–4 cell stage. At 72 hpf, the phenotype of the embryos was determined. (**b**) The cardiac phenotype was determined to be positive when the pericardium showed remarkable swelling over the tangent line of yolk and mandible. (**c**) The ratio of embryos with positive (black) and negative (white) phenotypes. The number of the animals is shown above each bar. (**d**) Representative animals of each group. No obvious phenotype was detected in any group.

**Figure 6:**
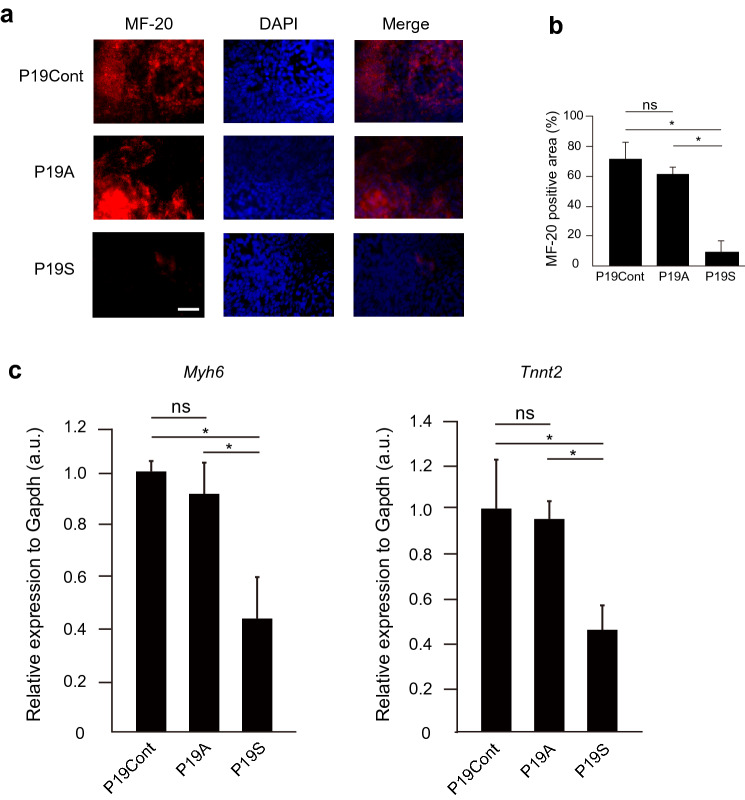
Accumulation, but not ablation of Smad9 attenuates cardiac differentiation. (**a**) Immunostaining of P19CL6 cells with anti MF-20 antibody after 12 days of induction of cardiac differentiation. The stable expression of Asb2 does not affect the cardiac induction while that of Smad9 suppress differentiation into cardiomyocytes. (**b**) MF-20 positive area. P19S shows siginificantly reduced MF-20 positive area. (**c**) The expression level of the common major cardiac marker *Myh6* and *TnnT2*. P19S showed decreased expression of both markers. The data are shown as the mean ± s.d. Statistical analysis was performed using one-way ANOVA with Tukey’s multiple comparison tests. *p < 0.05; ns, not significant.

To further explore the developmental function of ASB2 in vivo, we investigated cardiac development by employing zebrafish embryogenesis model. We injected a specific morpholino oligonucleotide (MO) directed to the AUG translational start site of zebrafish *Asb2*, resulting in suppression of transcription (A^AUG^-MO). Seventy-two hours post fertilization (hpf), marked swelling of the heart region was observed (Fig. 4a). We further examined another MO targeted against the splice donor and acceptor site of exon 7 of *Asb2* (A^SP^-MO) that leads to a frameshift resulting in premature transcript termination and produces a mutant lacking E3 ligase activity due to loss of the SOCS box domain or leads to collapsed expression through non-sense mediated decay. Similar to the A^AUG^-MO group, swelling of the heart region was observed in the A^SP^-MO specimen (Fig. 4b). Histological study revealed marked thinning of the ventricular wall (Fig. 4c) and dilation of the ventricular diameter (Fig. 4d) in both the A^AUG^- and A^SP^-MO groups compared to wildtype siblings (8.7 ± 0.9 versus 4.6 ± 0.8 and 3.6 ± 0.8, 40.1 ± 1.5 versus 54.6 ± 2.3 and 56.7 ± 2.7; *p* < 0.05, respectively). Of note, the significant phenotype was detected only in the heart in embryos at 72 hpf, not in the other parts of the body, including skeletal muscle (Fig. 4b, bottom), consistent with cardiac-specific expression in early embryogenesis (Fig. 3a). Next, we hypothesized that the cardiac phenotype of *Asb2* knockdown by MO was the consequence of Smad9 accumulation due to loss of its E3 ligase Asb2. To test this hypothesis, we simultaneously knocked down zebrafish *Smad9* with *Asb2*. An MO targeting the AUG translational start site of *Smad9* (S^AUG^-MO) was injected with or without A^AUG^-MO (Fig. 5a). We defined a positive phenotype as pericardium showing remarkable swelling over the tangent line of yolk and mandible (Fig. 5b). As expected, simultaneous knockdown of both *Asb2* and *Smad9* partially rescued the phenotype of A^AUG^-MO (Fig. 5c,d), indicating that quantitative suppression of *Smad9* transcripts ameliorated the accumulation of Smad9 protein induced by loss of its E3 ligase, Asb2. We further confirmed this concept using P19CL6 cells because P19CL6 cells possess the differential capacity of cardiomyocytes that is reported to depend on the BMP signaling^27^. We established P19CL6 cells that stably express either Asb2 (P19-A) or Smad9 (P19-S). After cardiac induction, P19-A showed comparable MF-20 positive area, whereas P19-S showed significantly smaller area than the control (Fig. 6a,b). Cardiac specific markers such as *Myh6* and *Tnnt2* also showed lesser expression only in P19-S (Fig. 6c). Collectively, these results suggest that accumulation of Smad9 attenuates normal cardiac development, and simultaneous expression of Asb2 is required to avoid excessive accumulation of Smad9.

### ASB2 regulates Tbx2 expression through SMAD9 degradation

**Figure 7:**
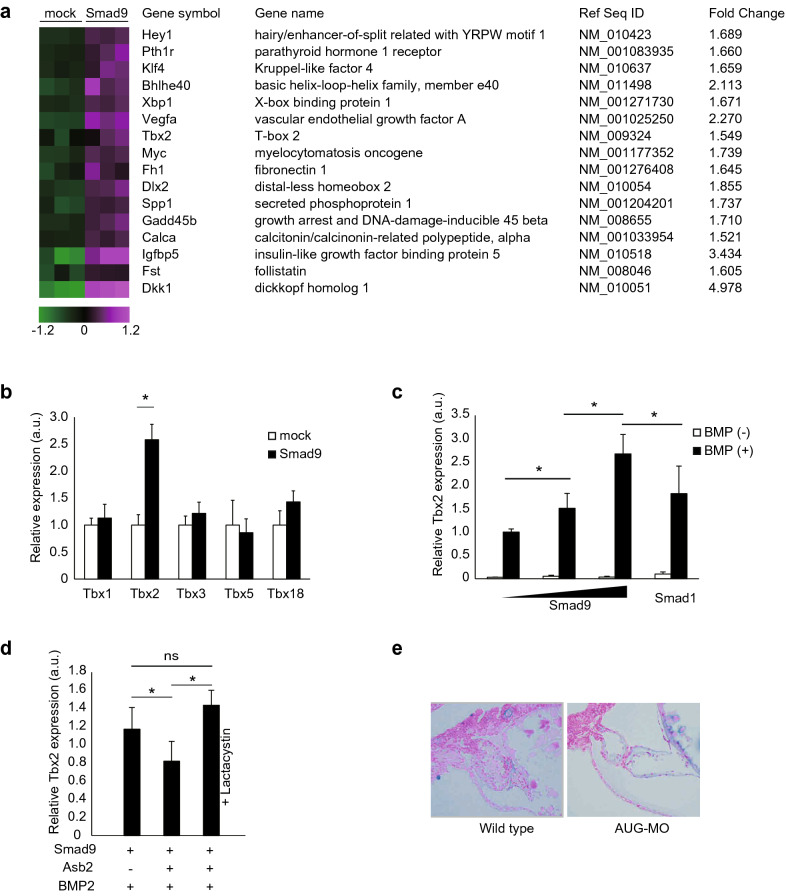
Tbx2 expression is regulated by SMAD9. (**a**) Search for SMAD9 target genes through comprehensive gene expression analysis. P19CL6 cells were transfected with either the mock vector or the vector coding Flag-SMAD9 and treated with 100 µM BMP2 for 24 h. RNA was collected and subjected to microarray analysis. Sixteen BMP-responsive genes were significantly upregulated by forced expression of SMAD9. (**b**) P19CL6 cells were transfected with either the mock vector or the vector encoding Flag-SMAD9 and treated with 100 µM BMP2 for 24 h. The expression levels of indicated genes were determined using quantitative real-time PCR. Among Tbx family, only Tbx2 was significantly induced by forced expression of SMAD9 under BMP2 stimulation. (**c**) From the left, 0, 0.1, and 1.0 µg of vector encoding Flag-SMAD9 and 1.0 µg of vector coding Flag-SMAD1 were transfected into P19CL6 cells. The expression level of Tbx2 was positively correlated with the amount of SMAD9. Greater induction of Tbx2 was observed upon transfection of SMAD9 compared to SMAD1. (**d**) Tbx2 expression was induced by treatment with BMP2. The expression level of Smad9 was blunted by co-expression of ASB2. The blunting effect of ASB2 was cancelled by treatment with 10 µM lactacystin for 16 h. (**e**) The histological section of zebrafish was subjected to alcian blue staining to visualize excess extracellular matrix induced by misexpression of Tbx2. The data are shown as the mean ± s.d. Statistical analysis was performed using one-way ANOVA with Tukey’s multiple comparison tests. *p < 0.05; ns, not significant; a.u., arbitrary unit.

To understand how the accumulation of SMAD9 attenuates normal cardiac development, it is critical to reveal the target gene of SMAD9 because the primary biological function of R-SMADs is the transduction of the BMP signal into the nucleus through induction of expression of its gene targets as transcription factors. However, to the best of our knowledge, the specific target genes of SMAD9 have not been reported. It is widely accepted that transcription of the target gene is regulated by a co-expressed factor that mediates the binding of transcription factors and genomic DNA^28^. P19CL6 cells possess the deifferential capacity of cardiomyocytes^27^ and this differentiation is reported to depend on the BMP signalling. Thus, we decided to employ P19CL6 cells to effectively explore the possible target genes involved in ASB2-mediated regulation of BMP signalling through SMAD9 ubiquitylation in developing heart. First, to search for genes whose expression is upregulated by SMAD9 accumulation, we transfected either the vector expressing Flag-SMAD9 or a mock vector into P19CL6 cells and treated them with BMP2 for 24 h under serum-free conditions. The collected RNA samples were subjected to microarray analysis. The data demonstrated that 268 probe sets were significantly upregulated more than 1.5-fold in the SMAD9-transfected cells. We next analysed these candidates using Ingenuity Pathway Analysis to identify genes known to be regulated by BMP signalling. Finally, we obtained 16 candidate genes as SMAD9 targets (Fig. 7a). Among them, Tbx2 elicited great interest because Tbx2 is both necessary and sufficient to suppress ventricular chamber differentiation^29,30^, which can give plenary explanation to the phenotype in zebrafish. To confirm the induction of Tbx2 by SMAD9, we checked the expression levels of Tbx genes in BMP2-treated P19CL6 cells with or without SMAD9 overexpression (Fig. 7b). qRT-PCR showed that Tbx2 was significantly upregulated by co-transfection of SMAD9, whereas other Tbx genes were not. Additionally, the expression of Tbx2 is positively correlated with SMAD9 expression in a dose-dependent manner (Fig. 7c). Moreover, the same amount of SMAD9 induced Tbx2 more than SMAD1. Next, to investigate whether Tbx2 expression is affected by ASB2 via SMAD9 regulation, we checked Tbx2 expression in P19CL6 cells co-transfected with vectors encoding ASB2 and SMAD9. Our results indicated that Tbx2 expression due to BMP2 treatment was upregulated by forced expression of SMAD9 and was suppressed by co-expression of ASB2, and was rescued by treatment with lactacystin (Fig. 7d). These data suggest that knockdown of ASB2 leads to SMAD9 accumulation and consequent induction of Tbx2. Previous studies have revealed that overexpression of Tbx2 in the myocardium suppresses the cardiac differentiation accompanying abnormal deposition of acidic glycosaminoglycan, an extracellular matrix component stained by alcian blue^31^. Therefore, to test whether Tbx2 induction causes the cardiac phenotype shown in the zebrafish model, we conducted alcian blue staining in the zebrafish model. The histological section of the zebrafish heart revealed that glycosaminoglycan was abnormally deposited in ventricles in the A^AUG^-MO treated group, which also featured dilated ventricular diameter and thinned ventricle walls (Fig. 7e). These data suggest that SMAD9 induces the expression of Tbx2 in developing hearts and that ASB2 regulates the function of Tbx2 through quantitative regulation of SMAD9.

## Discussion

BMP signalling is the most primitive and fundamental signal during embryogenesis and requires balanced dynamic tuning with temporal activation and shutdown^18,32–34^. Selective and aggressive degradation of the intracellular R-SMAD signal transducers by the UPS is one of the most important negative regulatory machineries of BMP signalling^18,20,35,36^. However, the unique E3 ligase of SMAD9 has not been identified yet. Here, we identified ASB2 as the E3 ligase of SMAD9 for the first time. Our results revealed the importance of the quantitative negative regulation of SMAD9 by its E3 ligase ASB2 during embryogenesis, specifically in the developing heart.

So far, several studies have reported that the E3 ligase SMURF 1/2 ubiquitylates R-SMADs of the TGFβ superfamily, including BMP, and specifically accelerates their degradation^18–20,23,35,36^. However, neither ubiquitylation nor proteasomal degradation of SMAD9 mediated by SMURF 1/2 has been conclusively shown. Additionally, Shi et al. have shown that Smurf1 reduces protein levels of Smad1 and Smad5 but not Smad9 in cultured lung explants^23^, suggesting that SMAD9 escapes from the quantitative regulation by Smurf 1/2 and potentially has its unique E3 ligase independently from Smad1/5. Our in silico search for SMAD9-binding proteins found two novel candidate E3 ligases including ASB2 (Fig. 1a,b). We confirmed that ASB2 interacts with SMAD9 as its E3 ligase (Fig. 1b,c) and target SMAD9 protein to proteasomal degradation (Fig. 1d,e). Furthermore, we demonstrated that SMAD9 is predominantly ubiquitilated by ASB2 while other R-SMADs are ubiquitilated by Smurf1/2 (Fig. 2). This specificity could be explained by the sequence homology and divergence; SMAD1, 5 and 9 share two highly conserved functional domains, MH1 and MH2, which lead to the functional redundancy among them in the various biological functions (Supplemental Fig. S2). The MH1 domain on the N-terminal side is required for DNA binding, whereas the MH2 domain on the C-terminal side is responsible for protein binding with BMP receptors^37^. On the other hand, however, sequence divergence exists in the linker region between these two domains and is believed to be largely responsible for the specific function of each R-SMAD^38,39^. In particular, SMAD9 is phylogenetically derived from SMAD1/5, and its linker region shares fewer amino acids with those of SMAD1/5^26,40^. Additionally, SMAD9 does not contain the PPYP motif that is required for interaction with SMURF1/2 and conserved in the linker region of all the other R-SMADs^38^. These facts suggest that, evolutionally, SMAD9 was spared from SMURF-mediated negative regulation by the lack of a PPYP motif and is instead uniquely regulated by ASB2 to achieve appropriate control of BMP signalling when it should be downregulated. In this context, it is important to define the ASB2 binding site of SMAD9. Recently, great efforts have sought to elucidate the importance of linker phosphorylation of R-SMADs triggered by mitogen-activated protein kinases (MAPKs) and cyclin-dependent kinases (CDKs)^39^. Several studies have revealed that linker phosphorylation is critical for the recognition of R-SMADs by other proteins, including Smurf 1/2^38,41,42^. It is possible that similar regulation could control SMAD9 recognition by ASB2. Deeper insight into such a spatiotemporal specificity of the ASB2-SMAD9 interaction will allow a more precise understanding of the mechanisms of early cardiogenesis.

ASB2, a newly identified E3 ligase of SMAD9, was originally identified as a retinoic acid-inducible protein in myeloid leukaemia cells in which Asb2 is regulated by PML-RARα, a pathological gene sequence generated because of translocation t(15;17)^43^. In the physiological condition, ASB2 is reported to recruit Filamin A and Filamin B as its substrates and regulate the differentiation of the hematopoietic cells and myogenic development of C2C12 cells^44,45^. Just recently, it was also reported that degradation of Filamin A by Asb2 is required for cardiogenesis^46,47^. These studies demonstrated that deletion of Asb2 results in preserved Filamin A protein that enhances TGFβ signalling through direct interaction with Smad2. However, the deletion of Filamin A could not completely rescue the phenotype of Asb2 deletion, suggesting existence of alternative mechanisms which leads to abnormal cardiac developmental phenotype due to deletion of Asb2. Consistent with these reports, our data here demonstrated that cardiac phenotype due to Asb2 knockdown was partially rescued by simultaneous knockdown of its substrate Smad9, indicting accumulation of either of Smad2 or Smad9 may attenuate normal cardiac development. Of note, Asb2 does not target Smad2 for degradation by UPS (Fig. 2), indicating Smad2 and Smad9 contribute to cardiac development independently each other. Previous reports also presented cardiac specific expression of Asb2 during embryogenesis from E8 or E9.5. Here, we provided more detailed evidence showing that Asb2 expression is highly specific to the cardiac region from earlier phase, as early as cardiac crescent formation at E7.5 (Fig. 3), Asb2 expression was solely detected in the cardiac region until completion of heart formation in murine embryo, suggesting more critical involvement of Asb2-Smad9 axis in the cardiogenesis. Actually, MO-driven ablation of Asb2 resulted in reduced cardiac cells and dilated ventricle in zebrafish embryo (Fig. 4). This cardiac phenotype produced by *Asb2* knockdown in zebrafish embryos was partially rescued by simultaneous knockdown of *Smad9* (Fig. 5), suggesting that Smad9 degradation mediated by Asb2 is at least partially involved in normal development of the heart. We could not exclude the possibility that ASB2 also ubiquitylates other proteins including Filamin A to exert its function, that could possibly explain the reason of partial rescue of the zebrafish phenotype (Fig. 5). Additionally, the expression of ASB2 remained constant in adult hearts, whereas SMAD9 expression in the heart was suppressed in later stages of embryogenesis^26,48^. Because it is quite possible that ASB2 might recruit other substrates in adult myocardium, further investigation of these hypotheses is of great interest.

Previous studies using knockout mice showed that *Smad9* homo- and heterozygotes are viable and fertile, showing no obvious abnormal phenotype^26^ or limited phenotypical differences in the pulmonary artery^48^. The lack of a critical *Smad9*-depleted phenotype may be explained by the redundancy between Smad9 and Smad1/5. However, the phenotype of SMAD9 accumulation, not deletion, is still unveiled. We demonstrated that abundant SMAD9 protein suppresses differentiation of cardiomyocyte while overexpression of its E3 ligase ASB2 did not affect the cardiac differentiation in vitro (Fig. 6), suggesting that accumulation, but not deletion of SMAD9 affect the normal cardiac development, that is consistent with the results of experiments in zebrafish (Fig. 5). In the present study, to elucidate the physiological meaning of SMAD9 regulation, we investigated the target gene of SMAD9 in cardiogenesis and identified Tbx2. Previous studies using knockout mice showed that *Smad9* homo- and heterozygotes are viable and fertile, showing no obvious abnormal phenotype^26^ or limited phenotype differences in the pulmonary artery^48^. The lack of a critical *Smad9*-depleted phenotype may be explained by the redundancy between Smad9 and Smad1/5. However, the phenotype of SMAD9 accumulation, not deletion, is still unveiled. From our in vivo cardiogenesis experiment using zebrafish, E3-mediated quantitative regulation of Smad9 functions to prevent excess accumulation of Smad9. Why should SMAD9 accumulation be avoided in the developing heart? Our data demonstrated that one possible answer involves the inappropriate induction of Tbx2 (Fig. 7a,b). Forced expression of SMAD9 in P19CL6 cells, which have developmental potential to cardiomyocyte, resulted in upregulation of Tbx2. Under BMP signalling, SMAD9 upregulated Tbx2 but no other Tbx genes (Fig. 7b). Consistent with previous studies showing the BMP-regulated induction of the Tbx2 gene^31,49^, SMAD1, another R-SMAD of BMP signalling, could also induce the expression of the Tbx2 gene. However, the induction of the Tbx2 gene was positively correlated with SMAD9 expression and was more upregulated by SMAD9 compared to comparable expression of SMAD1 (Fig. 7c). These data suggest that SMAD9 plays a pivotal role in Tbx2 induction by BMP signalling. Tbx2 is a member of the T-box gene family and generally repress the cardiac chamber development. For example, Tbx2-null embryos show excess expression of chamber-specific genes in the atrio-ventricular canal^30^, whereas Tbx2-overexpression leads to downregulation of chamber-specific genes, resulting in hypoplastic myocardium^29,50,51^. Additionally, Tbx2 induces Has2 gene expression and increases hyaluronic acid (HA) deposition, which is required for endocardial cushion formation. However, misexpression of Tbx2 in the myocardium results in HA deposition, which prevents normal cardiac differentiation^31^. Consistent with these previous reports, our present data collected from alcian blue staining clearly showed the accumulation of HA in the *Asb2* knockdown model in which Smad9 accumulation and consequent tbx2 induction might occur (Fig. 7e). Collectively, we showed for the first time that ASB2 is a novel and unique E3 ligase of SMAD9 and temporal quantitative regulation of SMAD9 by ASB2 attenuates BMP signalling and play pivotal roles in cardiogenesis.

## Methods

### Reagents and antibodies

The following antibodies were purchased from the indicated sources: HRP-conjugated anti-Flag-M2 (Sigma, 1:1000 immunoblotting (IB)), HRP-conjugated anti-hemagglutinin (HA)-tag (Sigma, 1:500 IB), HRP-conjugated anti-Myc (Santa Cruz Biotechnology, 1:1000 IB), and Anti-Actin (MBL, 1:3000 IB). HRP-conjugated anti-rabbit IgG (1:5000 IB) was purchased from Cell Signaling. For immunoprecipitation, anti-FLAG-M2 affinity gel (Sigma, 10 μL immunoprecipitation (IP)) and C-Myc monoclonal antibody-agarose beads (Clontech, 10 μL IP) were used. The following reagents were purchased from the indicated sources: recombinant hBMP2 (Millipore, 100 µM), lactacystin (Sigma, 10 µM), and blasticidin S (Wako, 10 µg mL^-1^).

### Plasmids

Plasmid constructs of Myc-tagged Smurf1 and Smurf2 were generated using the Gateway System (Life Technologies) according to the manufacturer’s protocol. The pcDEF-Flag Smad1, Smad2, Smad3, Smad5 and Smad9 were kindly provided by Dr. Miyazono (University of Tokyo). DNA fragments encoding Flag-tagged Smad9, HA-tagged DNAJA3 and HA- or Myc-tagged Asb2 flanking the recognition sites of proper restriction enzymes were amplified using PCR with Multiple Tissue Panels of cDNA (Clontech) as template and sub-cloned into the pcDEF vector. The sequences of the obtained plasmids were confirmed using DNA sequencing. The Xvent2-Luc vector was kindly provided by Dr. Toshihiko Ogura (Tohoku University).

### Cell culture and transfection

P19CL6 cells were provided by the RIKEN BRC through the National Bio-Resource Project of the MEXT (Japan). P19CL6 cells were maintained in alpha-MEM supplemented with 10% foetal bovine serum (FBS) and 1% penicillin/streptomycin. HEK293 and HeLa cells were obtained from the ATCC and cultured in DMEM supplemented with 10% FBS and 1% penicillin/streptomycin. Cells were transfected with FuGene HD (Promega) according to the manufacturer’s protocol. Stable cell lines were generated by transfecting the plasmid coding either of ASB2 or SMAD9 together with linearized puromycin resistant cassette followed by limiting dilution culture under presence of puromycin. Single colony was picked up and cultured to obtain isolated clones.

### Immunoprecipitation and western blotting analysis

After washing with PBS, cells were harvested with lysis buffer (1% CHAPS, 150 mM NaCl, 20 mM MOPS, 1 mM EDTA, 10% glycerol, and protease inhibitor cocktail (Nakalai Tesque)). Protein concentration was measured, and 10 µg of proteins was separated using SDS-PAGE. For immunoprecipitation assays, 1 mg of proteins was incubated with 10 µL of the indicated beads overnight at 4 °C. After extensive washing with lysis buffer, the supernatant was discarded, and the immunocomplexes remaining on the beads were separated with SDS-PAGE. After transfer to polyvinylidene difluoride membranes (Millipore) and blocking with 3% skim milk in TBS for 30 min, immunoblotting was performed with the indicated antibodies for 1 h at room temperature. For the detection of actin, an additional wash with TBS was followed by immunoblotting with secondary antibody for 1 h at room temperature. After blotting, membranes were washed three times with TBS and incubated with ECL Prime (Sigma). Protein bands were visualized using a Bio-Rad ChemiDoc XRS System (Bio-Rad Laboratories).

### Pulse-chase analysis

HEK293 cells were transfected with the indicated plasmids. Twenty-four hours after transfection, cycloheximide was added at a final concentration of 50 µg/mL to terminate further protein synthesis. Cells were harvested at 0, 1, 2, 4, 6 and 8 h after the addition of CHX and subjected to western blotting assay. Protein levels were quantified from the band density using Bio-Rad Quantity One 1-D Analysis software (Bio-Rad Laboratories).

### Luciferase assay

Luciferase assays were conducted using a Dual-Glo Luciferase Assay System (Promega). Briefly, HeLa cells were transfected with both an Xvent2-Luc plasmid encoding the firefly luciferase gene under the Xvent2 promoter and a PGL4.75 vector (Promega) encoding *Renilla* luciferase under the CMV promoter as a control. Simultaneously, vectors encoding Flag-Smad9, Flag-Smad4 and Myc-Asb2 were transfected. Immediately after the transfection, cells were treated with or without 100 µM BMP2. After 24 h, cells were washed twice with PBS and treated with Dual-Glo Luciferase Assay Reagent diluted by half with PBS. After incubating for 10 min at room temperature, luminescence was measured for firefly luciferase activity from the Xvent2 vector. Then, Dual-Glo Stop & Glo Reagent was added, and the sample was incubated for another 10 min at room temperature. Finally, luminescence was measured for *Renilla* luciferase activity from the *Renilla* luciferase vector. To normalize the data, the firefly:*Renilla* luminescence ratio was calculated for each sample.

### RT-PCR and Real-time PCR

To quantitate gene expression levels, RNA was extracted using TRIZOL reagent (Sigma), and cDNA was obtained using a High-Capacity cDNA Reverse Transcription Kit (Thermo Fisher Scientific). The obtained templates were mixed with Taqman Fast Universal PCR Master Mix (Thermo Fisher Scientific) and the assay mix (Thermo Fisher Scientific) and subjected to real-time reverse transcription (RT)-PCR with an ABI Fast Real-Time PCR System. The relative expression level was calculated using the ∆∆CT method.

### Animals

All procedures were conducted in compliance with the ARRIVE guidelines and with the Guide for the Care and Use of Laboratory Animals of National Cerebral and Cardiovascular Center. The animal protocol was approved by the Ethical Committee of National Cerebral and Cardiovascular Center and Osaka University Committee for Laboratory Animal Use.

### Whole-mount in situ hybridization

Whole-mount in situ hybridization was performed as described previously^31^. Briefly, staged mouse embryos were obtained and fixed in MEMFA (0.1 M Mops/2 mM EGTA/1 mM MgSO_4_/3.7% formaldehyde). The fixed embryos were stored in 90% methanol at − 20 °C until use for hybridization. Whole-mount in situ hybridization was conducted as described by Yamada et al. ^49^ using digoxin-UTP-labelled RNA probes according to the manufacturers’ protocols. Probes were cDNAs designed for mouse Asb2.

### Zebrafish experiments

General maintenance, collection and staging of the zebrafish were carried out according to established convention. At cell stages 1–4, 3 or 6 ng of MO or mock vector was injected into zebrafish embryos. All MOs were synthesized using Gene-Tools. The sequences of the MO were as follows; A^AUG^-MO: TTCGGCATAAGAGAACCGGGTCATC, A^SP^-MO: GTTGCTGATGAGACTCACAGGTCTT, S^AUG^-MO: TGCATCGTGAAACGGGTTGATTTTA and A^SP^-MO . The injected zebrafish embryos were sacrificed at 72 hpf and fixed in 10% formamide. Haematoxylin–eosin stained sample slides of sagittal sections were made by Applied Medical Research (Japan).

### Microarray analysis

For microarray analysis, P19CL6 cells were transfected with a plasmid encoding Flag-Smad9 or mock vector (n = 3 each). Twenty-four hours after transfection, the cells were treated with 100 µM BMP2 for an additional 24 h, and then RNA samples were collected. Gene expression was determined using the Mouse Genome 430 2.0 Affymetrix GeneChip. The collected data were analysed using GeneSpring7 software (Agilent Technologies). First, expression data were normalized using the robust multi-array average (RMA) approach. We eliminated probe sets that were absent from all the samples and selected those that demonstrated *p* < 0.05 according to the Mann–Whitney U test. Among the remaining probe sets, 268 probe sets were upregulated by 1.5-fold or more in Smad9-transfected cells compared with mock-transfected cells. Next, we analysed probe sets using Ingenuity Pathway Analysis (Ingenuity) and selected genes known to be regulated by BMP signal activation.

### Statistics

The results of RT-PCR, the luciferase assay, and ventricular wall thickness and ventricular diameter measurements in zebrafish histology are represented as the mean ± s.d. The Shapiro–Wilk normality test showed the data distributed normally unless noted otherwise. Statistical analysis were performed by one-way ANOVA with Tukey’s multiple comparison tests to test > 2 groups. Statistical significance was defined when *p < 0.05.

## Supplementary Information

Supplementary Information 1.

Supplementary Information 2.

Supplementary Information 3.

Supplementary Information 4.
