# Supplementary material for: ASB2 is a novel E3 ligase of SMAD9 required for cardiogenesis
Source: Sci Rep. 2021 Nov 29;11:23056. doi: 10.1038/s41598-021-02390-0 (PMC8630118; doi:10.1038/s41598-021-02390-0)

**Supplementary informantion**

**ASB2 is a novel E3 ligase of SMAD9 required for cardiogenesis**

Kyung-Duk Min^1^, Masanori Asakura^1,2*^, Manabu Shirai^3,4^, Satoru Yamazaki^5,6^, Shin Ito^1^, Hai Ying Fu^1,7^, Hiroshi Asanuma^8^,　Yoshihiro Asano^9^, Tetsuo Minamino^7^, Seiji Takashima^10^, Masafumi Kitakaze^1,11*^

^1^ Department of Clinical Research and Development, National Cerebral and Cardiovascular Center, Osaka, Japan.^2^ Department of Cardiovascular and Renal Medicine, Hyogo College of Medicine, Hyogo, Japan. ^3^Department of Bioscience, National Cerebral and Cardiovascular Center, Osaka, Japan. ^4^ Omics Research Center, National Cerebral and Cardiovascular Center, Osaka, Japan. ^5^ Department of Cell Biology, National Cerebral and Cardiovascular Center, Osaka, Japan. ^6^ Department of Molecular Pharmacology, National Cerebral and Cardiovascular Center, Osaka, Japan. ^7^ Department of Cardiorenal and Cerebrovascular Medicine, Faculty of Medicine, Kagawa University, Kagawa, Japan. ^8^ Department of Internal Medicine, Meiji University of Integrative Medicine, Kyoto, Japan. ^9^ Department of Cardiovascular Medicine, Osaka University Graduate School of Medicine, Osaka, Japan. ^10^Department of Medical Biochemistry, Osaka University Graduate School of Medicine, Osaka, Japan. ^11^ Hanwa Daini Senboku Hospital, Sakai, Osaka, Japan.

*Corresponding author: Masafumi Kitakaze M.D., Ph.D., Department of Clinical Research and Development, National Cerebral and Cardiovascular Center, 6-1 Kishibe- Shimmachi, Suita, Osaka 564-8565, Japan., Tel.: +81-6-6170-1070; E-mail: kitakaze@zf6.so-net.ne.jp

Supplementary Figure S1


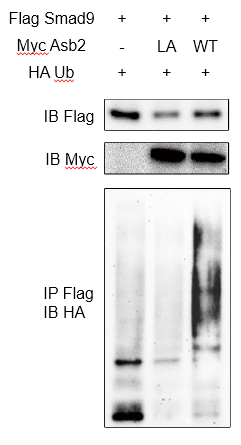


Supplementary Fig S1 A plasmid coding Smad9 was co-trasnfected with plasmids that codes either of wild type (WT) Asb2 or Asb2-LA that harbors a point mutation leading to loss of E3 activity. Asb2-LA failed to ubiquitinate Smad9 whilst Asb2-WT did as a control. The cropped gel images were delineated and uncropped gel images are shown in the Supplementary Information.

Supplemetary Figure S2

SMAD1 1 ----MNVTSLFSFTSPAVKRLLGWKQGDEEEKWAEKAVDALVKKLKKKKGAMEELEKALS
SMAD5 1 ---MTSMASLFSFTSPAVKRLLGWKQGDEEEKWAEKAVDALVKKLKKKKGAMEELEKALS
SMAD9 1 MHSTTPISSLFSFTSPAVKRLLGWKQGDEEEKWAEKAVDSLVKKLKKKKGAMDELERALS


SMAD1 57 CPGQPSNCVTIPRSLDGRLQVSHRKGLPHVIYCRVWRWPDLQSHHELKPLECCEFPFGSK
SMAD5 58 SPGQPSKCVTIPRSLDGRLQVSHRKGLPHVIYCRVWRWPDLQSHHELKPLDICEFPFGSK
SMAD9 61 CPGQPSKCVTIPRSLDGRLQVSHRKGLPHVIYCRVWRWPDLQSHHELKPLECCEFPFGSK


SMAD1 117 QKEVCINPYHYKRVESPVLPPVLVPRHSEYNPQHSLLAQFRNLGQN-EPHMPLNATFPDS
SMAD5 118 QKEVCINPYHYKRVESPVLPPVLVPRHNEFNPQHSLLVQFRNLSHN-EPHMPQNATFPDS
SMAD9 121 QKEVCINPYHYRRVETPVLPPVLVPRHSEYNPQLSLLAKFRSASLHSEPLMPHNATYPDS


SMAD1 176 FQQPNSHPFPHSPNSSYPNSPGSSSSTYPHSPTSSDPGSPFQMPADTPPPAYLPPEDPMT
SMAD5 177 FHQPNNTPFPLSPNSPYPPSP--ASSTYPNSPASSGPGSPFQLPADTPPPAYMPPDDQMG
SMAD9 181 FQQPPCSALPPSPSHAFSQSP--CTASYPHSPGSPS------------------------


SMAD1 236 QDGSQPMDTN-MMAPPLPSEINRGDVQAVAYEEPKHWCSIVYYELNNRVGEAFHASSTSV
SMAD5 235 QDNSQPMDTSNNMIPQIMPSISSRDVQPVAYEEPKHWCSIVYYELNNRVGEAFHASSTSV
SMAD9 215 ---------------EPESPYQHSDFRPVCYEEPQHWCSVAYYELNNRVGETFQASSRSV


SMAD1 295 LVDGFTDPSNNKNRFCLGLLSNVNRNSTIENTRRHIGKGVHLYYVGGEVYAECLSDSSIF
SMAD5 295 LVDGFTDPSNNKSRFCLGLLSNVNRNSTIENTRRHIGKGVHLYYVGGEVYAECLSDSSIF
SMAD9 260 LIDGFTDPSNNRNRFCLGLLSNVNRNSTIENTRRHIGKGVHLYYVGGEVYAECVSDSSIF


SMAD1 355 VQSRNCNYHHGFHPTTVCKIPSGCSLKIFNNQEFAQLLAQSVNHGFETVYELTKMCTIRM
SMAD5 355 VQSRNCNFHHGFHPTTVCKIPSSCSLKIFNNQEFAQLLAQSVNHGFEAVYELTKMCTIRM
SMAD9 320 VQSRNCNYQHGFHPATVCKIPSGCSLKVFNNQLFAQLLAQSVHHGFEVVYELTKMCTIRM


SMAD1 415 SFVKGWGAEYHRQDVTSTPCWIEIHLHGPLQWLDKVLTQMGSPHNPISSVS
SMAD5 415 SFVKGWGAEYHRQDVTSTPCWIEIHLHGPLQWLDKVLTQMGSPLNPISSVS
SMAD9 380 SFVKGWGAEYHRQDVTSTPCWIEIHLHGPLQWLDKVLTQMGSPHNPISSVS

Supplementary Fig S2 Protein sequence alignment of SMAD1/5/9. Multiple sequence alignment was examined with ClustalW (https://www.genome.jp/tools-bin/clustalw) and visualized with BoxShade (https://embnet.vital-it.ch/software/BOX_form.html).

Supplementary Figure S3


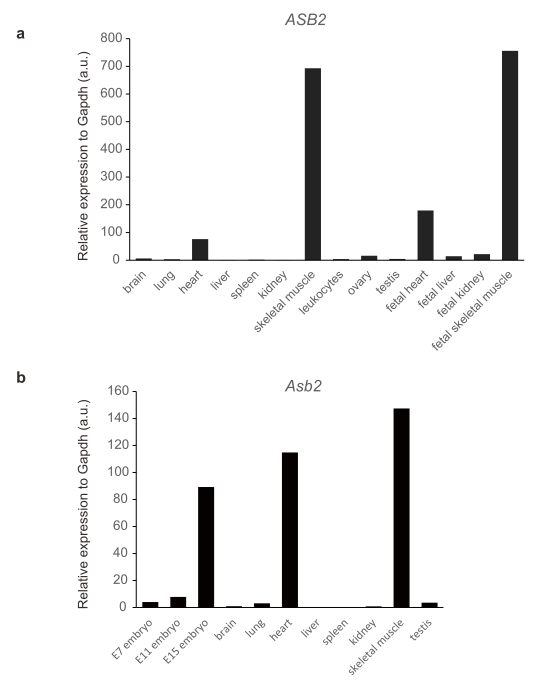


Supplementary Fig S3 Relative expression level of ASB2 gene in multiple tissues. ASB2 expression level in various tissues was determined using Multiple Tissue Panels for human and mouse (a and b, Takara Bio, Japan). The average of technically duplicated results of expression level was normalized to that of GAPDH.

Supplementary　Figure S4　　
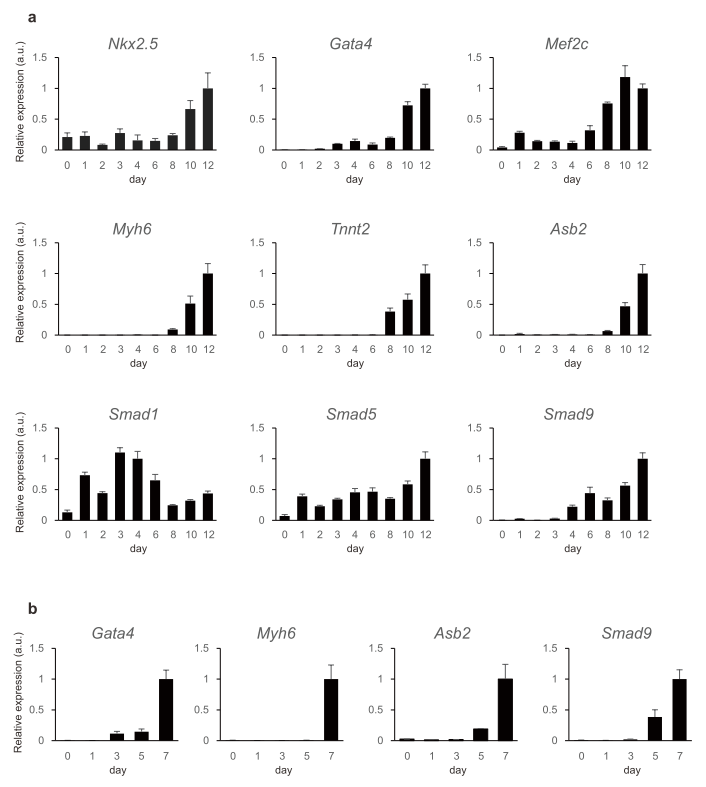


Supplementary Fig S4　Relative expression levels of cardiac specific genes during induction of cardiomyocytes. (a) P19CL6 cells were cultured with medium supplemented with 1% Dimethyl sulfoxide. (b) mouse ES cells were cultured using 96well low cell adhesion plate (PrimeSurface, Sumitomo BAKELITE, Japan) for 5days and then plated on 24 well plate coated with 2% gelatin-PBS. The mRNA were collected at indicated days and subjected to RT-PCR. Gene expression level was normalized to that of Gapdh.

Supplementary Figure S5


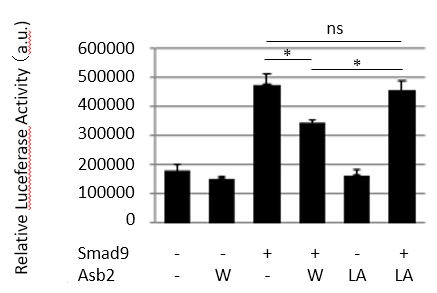


Supplementary Fig S5　Relative expression levels of luciferase activity. Smad9 was co-expressed with either of wild type Asb2 or Asb2-LA. Xvent2-Luc plasmid was also transfected and relative luciferase activity was measured after BMP2 stimulation.

Supplementary Table Legends

(The title and the legend are also included in each Supplementary Table provided as MS Excel spreadsheet.)

Supplementary Table S1. Candidates for SMAD9 interacting proteins provided by MINT. The table was downloaded from the site of MINT (<http://mint.bio.uniroma2.it/mint/Welcome.do>) as SMAD9 interacting proteins.

Supplementary Table S2. Candidates for SMAD9 interacting proteins provided by BioGRID. The table was downloaded from the site of BioGRID (<http://thebiogrid.org>) as SMAD9 interacting proteins.

Supplementary Table S3. Candidates for SMAD9 interacting proteins provided by previous study by Rual et al[^1^](#_ENREF_1). The Smad9 interacting proteins were picked up from the original database obtained from the supplementary information for indicated study by Rual et al.

Supplementary References

1. Rual, J.F.*, et al.* Towards a proteome-scale map of the human protein-protein interaction network. *Nature* **437**, 1173-1178 (2005).

Original full gel images of cropped gel images.

Figure 1b


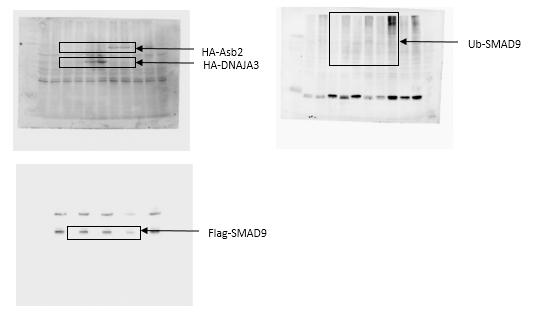


Figure 1c


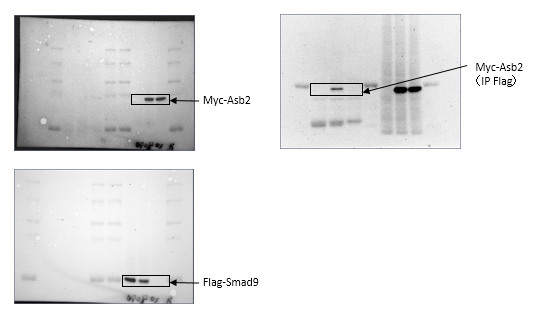


Figure 1d


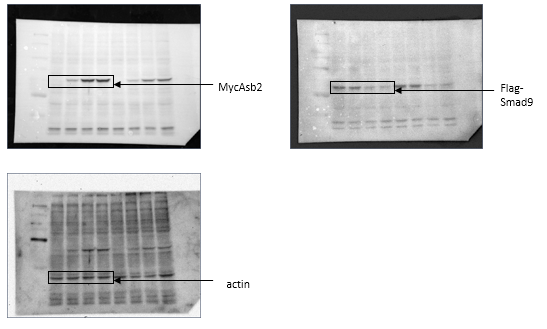


Figure 1e


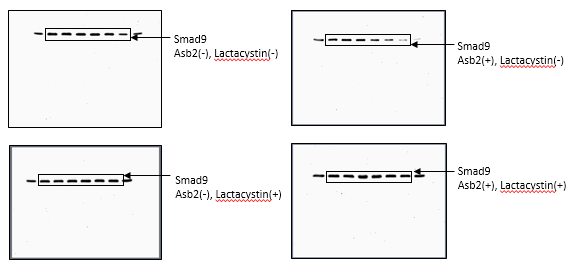


Figure 2


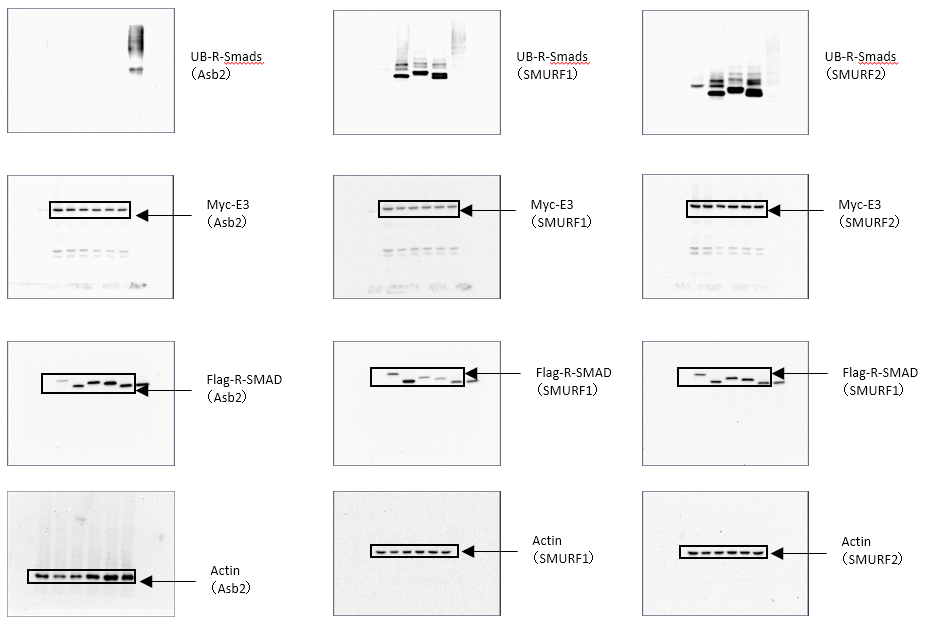


Supplemental Figure S1


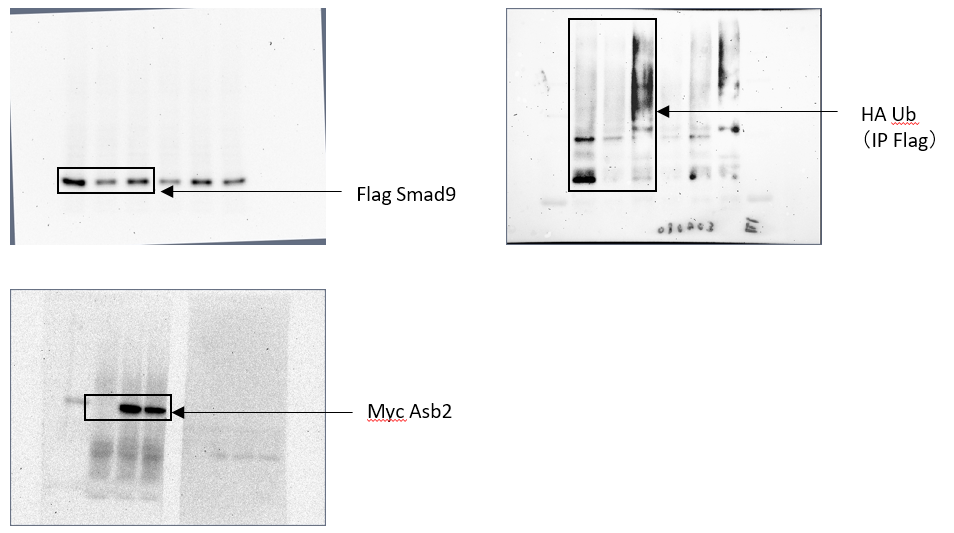

Supplement: Supplementary file 1 — Supplementary Information 1. [file 41598_2021_2390_MOESM1_ESM.docx]
